# Differential protein expression in human knee articular cartilage and medial meniscus using two different proteomic methods: a pilot analysis

**DOI:** 10.1186/s12891-018-2346-6

**Published:** 2018-11-29

**Authors:** Elin Folkesson, Aleksandra Turkiewicz, Martin Englund, Patrik Önnerfjord

**Affiliations:** 1 Department of Clinical Sciences Lund, Orthopaedics, Clinical Epidemiology Unit, Lund University, Faculty of Medicine, Lund, Sweden; 2 Department of Clinical Sciences Lund, Rheumatology and Molecular Skeletal Biology, Lund University, Faculty of Medicine, Lund, Sweden; 3 Clinical Epidemiology Research and Training Unit, Boston University School of Medicine, Boston, MA USA

**Keywords:** Meniscus, Articular cartilage, Proteomics, Knee, Data-independent acquisition, Data-dependent acquisition, Osteoarthritis

## Abstract

**Background:**

Proteomics is an emerging field in the study of joint disease. Our two aims with this pilot analysis were to compare healthy human knee articular cartilage with meniscus, two tissues both known to become affected in the osteoarthritic disease process, and to compare two mass spectrometry (MS)-based methods: data-dependent acquisition (DDA) and data-independent acquisition (DIA).

**Methods:**

Healthy knee articular cartilage taken from the medial tibial condyle and medial meniscus samples taken from the body region were obtained from three adult forensic medicine cases. Proteins were extracted from tissue pieces and prepared for MS analysis. Each sample was subjected to liquid chromatography (LC)-MS/MS analysis using an Orbitrap mass spectrometer, and run in both DDA and DIA mode. Linear mixed effects models were used for statistical analysis.

**Results:**

A total of 653 proteins were identified in the DDA analysis, of which the majority was present in both tissue types. Only proteins with quantitation information in both tissues (*n* = 90) were selected for more detailed analysis, of which the majority did not statistically significantly differ in abundance between the two tissue types, in either of the MS analyses. However, 21 proteins were statistically significantly different (*p* < 0.05) between meniscus and cartilage in the DIA analysis. Out of these, 11 proteins were also significantly different in the DDA analysis. Aggrecan core protein was the most abundant protein in articular cartilage and significantly differed between the two tissues in both methods. The corresponding protein in meniscus was serum albumin. Dermatopontin exhibited the highest meniscus vs articular cartilage ratio among the statistically significant proteins. The DIA method led to narrower confidence intervals for the abundance differences between the two tissue types than DDA.

**Conclusions:**

Although articular cartilage and meniscus had similar proteomic composition, we detected several differences by MS. Between the two analyses, DIA yielded more precise estimates and more statistically significant different proteins than DDA, and had no missing values, which makes it preferable for future LC-MS/MS analyses.

**Electronic supplementary material:**

The online version of this article (10.1186/s12891-018-2346-6) contains supplementary material, which is available to authorized users.

## Background

Osteoarthritis (OA) is a chronic joint disease, traditionally characterised by loss of articular cartilage. However, for the knee, in the last years, more and more interest has also been directed towards the meniscus since meniscal damage is strongly associated with development of knee OA [1]. Both articular cartilage and menisci have similar functions, which are to withstand load and to distribute weight across surfaces, but their ultrastructure is somewhat different [2, 3]. Articular cartilage consists of chondrocytes that produce structural macromolecules, which, together with water, builds up the extracellular matrix (ECM) that surrounds the chondrocytes [4]. These macromolecules are mainly collagens (predominantly type II) and proteoglycans (predominantly aggrecan) as well as non-collagenous proteins and glycoproteins. The meniscus also contains a dense ECM, but unlike articular cartilage, which only has one cell-type, the meniscus contains several different cell-types in its different regions [5, 6]. In the outer (peripheral) parts of the meniscus, which also contains blood vessels (red zone), the cells are mostly elongated fibroblast-like and in the inner region (white zone), the cells are mostly round chondrocyte-like. In addition, the superficial regions have been reported to host progenitor cells [5, 6].

Several histological techniques that allow analysis of the content and structure of articular cartilage and meniscus exist, such as immunohistochemistry, autoradiography or various types of tissue staining, e.g. haematoxylin and eosin staining [7]. However, the majority of these methods only provide information about macromolecular structures in the tissue or allow single-protein detection and analysis. During the last decades, new techniques, such as mass spectrometry (MS)-based proteomics, have allowed a more comprehensive analysis of a wide variety of tissues including cartilage tissues [8]. MS coupled with liquid chromatography (LC) has become one of the most common methods to analyse protein content in complex samples. With non-targeted MS it is possible to identify several hundreds of proteins, even thousands, in one analysis [9, 10]. In the most commonly used approach in MS, called the bottom-up approach, proteins are digested into peptides, and the peptides are then separated by LC followed by ionization, separation and detection in the mass spectrometer and the resulting spectra are compared with theoretical spectra generated from a sequence database [11]. However, not all ions are selected for separation and subsequent detection. In data-dependent acquisition (DDA) mode, a selection of certain precursor ions is performed based on predefined criteria, e.g. the top *n* most intense ions identified in the first mass separation step (MS1) [12]. This is compared to data-independent acquisition (DIA), where all precursor ions within a certain mass-to-charge ratio (m/z) range (larger windows of 20-25 Da width) are selected for fragmentation and the second mass analysis step (MS2) [9, 13]. Even though DIA has become increasingly utilised, DDA still is the preferred MS method. Thus, our aim was to compare human menisci with human articular cartilage using both DDA and DIA to identify biological differences and select the best methodological approach for future studies.

## Methods

### Patients and tissue material

Macroscopically normal knee articular cartilage and meniscus samples were obtained from forensic medicine cases at the University Hospital, Oslo, Norway. The collection was approved by the local ethics committee. Articular cartilage and medial menisci from three donors, aged 36 (male), 43 (male), and 41 (female) years with no history of joint disease, were included. Approximately 1 × 1 cm large knee articular cartilage pieces were taken perpendicular to the cartilage surface from the medial tibial condyle (representing full-depth cartilage). Full-depth tissue pieces of the medial meniscus were taken from the body region, including both inner and outer regions (synovium and fat was removed).

### Materials

*N*-Ethylmaleimide, 6-aminocaproic acid, benzamidine hydrochloride hydrate, dithiothreitol (DTT), iodoacetamide, ammonium bicarbonate (AMBIC), formic acid, HPLC grade acetonitrile and A (0.1% formic acid in water) and B (0.1% formic acid in acetonitrile) solutions for LC-MS were purchased from Sigma-Aldrich (St. Louis, USA). Guanidine hydrochloride (GdnHCl) and anhydrous sodium acetate (NaAc) were purchased from Merck (Darmstadt, Germany). Trypsin gold MS grade was purchased from Promega (Madison, WI). The water used in this study was purified using a MilliQ apparatus (Millipore, Billerica, MA).

### Preparation of tissue

For a schematic representation of the sample preparation process, please see Additional file 1: Figure S1*.* The dissected tissue was frozen (− 80 °C) and pulverized in liquid nitrogen using a ball grinder, after which the pulverized tissue was weighed. The proteins were extracted from the pulverized tissue using 15 volumes of chaotropic buffer (4 M GdnHCl, 50 mM NaAc, 100 mM 6-aminocaproic acid, 5 mM benzamidine, 5 mM *N*-ethylmaleimide, pH 5.8) for 24 h on an orbital shaker at + 4 °C. Extracts were collected after centrifugation at 13200 x g at + 4 °C for 30 min. The pellet was frozen and saved. Fifty μL of the extracts were reduced, using 4 mM DTT for 30 min shaking at + 56 °C. The extracts were alkylated using 16 mM iodoacetamide for 1 h at room temperature in the dark. In order to remove residual salts the extracts were precipitated with nine volumes of ethanol for 4 h at − 20 °C, after which the precipitate was dried in a SpeedVac and suspended in 100 μL of 0.1 M AMBIC, pH 8.5. The samples were then digested using 2 μg trypsin gold on a shaker at + 37 °C for approximately 16 h. The peptide concentrations of the digests were determined using Pierce Quantitative Colorimetric Peptide Assay (Thermo Fisher Scientific, Rockford, USA) according to the manufacturer’s instructions. Samples (50 μg) were diluted to 200 μL with a final concentration of 50 mM AMBIC and 0.5 M sodium chloride (to minimize ionic interactions). In order to remove peptides with glycosaminoglycan (GAG) chains from the samples, they were centrifuged through Nanosep® 30 K Omega Centrifugal Devices (Pall Life Sciences, Ann Arbor, USA. The samples were subsequently desalted and fractionated into two fractions (eluted with 10 and 50% acetonitrile respectively) using Pierce High pH Reversed-Phase Peptide Fractionation Kit (Thermo Fisher Scientific, Rockford, USA) according to the manufacturer’s instructions.

### Data-dependent acquisition

The digested samples were analysed using a quadrupole Orbitrap benchtop mass spectrometer, Q-Exactive, (Thermo Fisher Scientific). Two fractions from 5 μg digest of each sample were injected to an Easy nano-LC 1000 HPLC system (Thermo Fisher Scientific) equipped with an Acclaim PepMap® 100 nanoViper pre-column (Thermo Scientific, C18, 3 μm particles, 75 μm i.d. and 2 cm long) and an Acclaim PepMap® RSLC nanoViper analytical column (Thermo Scientific, C18, 2 μm particles, 75 μm i.d. and 25 cm long). A heated ion transfer setting of 260 °C was used for desolvation together with a spray voltage of + 2000 V. The on-line reversed-phase separation was performed using a flow rate of 300 nL/min. For the DDA analysis, a binary linear gradient of 85 min was used. The gradient started with 3% solvent B for 4 min, then going to 35% solvent B in 64 min, after which it goes to 45% solvent B in 5 min. Finally, the organic solvent concentration increased up to 90% in 5 min and kept at 90% for 7 min. The MS1 and MS2 scans were performed as previously described [14], with the exception of MS2 resolution that was set to 17,500 in this study. The system was controlled by Xcalibur™ Software (Thermo Fisher Scientific). Blank runs were injected between every sample to avoid cross-contamination. A spectral library was generated using the search result from one meniscus and one cartilage sample (4 DDA runs) in order to match the peptide retention times used for the DIA analysis.

### Data-independent acquisition

The instrumental set-up for the DIA analysis was almost the same as for the DDA analysis with some exceptions. In the DIA analysis, a longer gradient of 135 min was used, which started with 3% solvent B for 5 min, then increased to 35% solvent B in 120 min and then went up to 95% solvent B in 5 min. It ended with 95% solvent B for 5 min. For the MS settings, the MS1 scan (390-1210 m/z) was set to have a resolution of 70,000, 1 × 10^6^ automatic gain control (AGC) and 100 ms maximum ion injection time. This was followed by data-independent acquisition collision-induced dissociation MS2 scans at a resolution of 35,000, 1 × 10^6^ automatic gain control (AGC) and 120 ms maximum ion injection time. A loop count of 32 was used in the range 400-1200 m/z. The isolation windows were 26.0 m/z wide including 0.5 Da overlap.

### Data analysis

The raw DDA data were searched against the human Swiss-Prot database (Swiss-Prot_2015_06, containing 20,200 sequences) using Proteome Discoverer™ 2.1 Software (Thermo Fisher Scientific) as previously described [14], with the exceptions of included modifications, which in this study was: static modification: cysteine carbamidomethylation and dynamic modifications: N-terminal acetylation and methionine oxidation. In the data analysis, the top 3 peptides are averaged, which is based on a study that reported that the average MS signal response for the three most intense tryptic peptides signals per mole of protein is constant [15]. The peak intensity reported represents the abundance of the protein in the tissue extracts. The spectral library used was created in Proteome Discoverer™ 2.1 using the four DDA runs that were run using the same gradient as the DIA runs and imported into Skyline Daily (MacCoss Laboratories). The DIA data was analysed in Skyline and matched against the generated spectral library. Only multiple-charged (2,3) precursor ions and single-charged fragment ions were included, together with default ion types (b, y). N-terminal to proline was used as special ions, and the ion match tolerance was set to 0.1 m/z. Since the aim of this study was to compare the DDA and DIA protocol to select the best approach for further studies of samples from OA, we chose to narrow down the number of proteins included in the comparison. Therefore, only a subset of the identified proteins was selected for analysis. This selection was based on the DDA data by performing several filtration steps on the data in Proteome Discoverer™ 2.1 according to the following criteria: (1) proteins had to have at least two unique peptides per protein, (2) proteins had to be classified as an ECM protein (GO accession term: 0031012) and (3) proteins had to be identified in both tissue types. The data analysis of the DIA analysis was focused on the proteins remaining after these filtration steps. Manual peak selection of the peptides was performed in Skyline and remaining proteins and peptides were the basis of the statistical analysis. After the peak selection 103 proteins remained (see Additional file 2: Table S1).

### Statistical analysis

Only MS1 data from the DIA and DDA analysis was included in the statistical analysis which was performed in Stata (Release 14, StataCorp, 2015). All peak area intensity values for each precursor ion were added together, resulting in one peak area value for each protein in each fraction. In order to get one peak area value for each patient, the two fractions for each sample were added together and this value was used for further analysis. By selecting the top 3 peptides for each protein in the DIA analysis and calculating a mean value, we received a peak area value comparable to the DDA data. Only proteins with quantitative information in all patients from both tissues (*n* = 90 proteins) were included in the statistical analysis. Data was analysed using a linear mixed-effect model fitted through restricted maximum likelihood using the ANOVA method for computing degrees of freedom. The tissue donor was included as a random effect and the tissue (meniscus or articular cartilage) was included as fixed effect. Data was log2 transformed before the analysis. To mimic a typical analysis of data in proteome studies, we also performed an analysis with a control of the false discovery rate (FDR), using the method of Benjamini and Hochberg [16]. We considered a two-tailed *p*-value less or equal to 0.05 to be statistically significant.

## Results

### Protein identification based on DDA

**Fig. 1:**
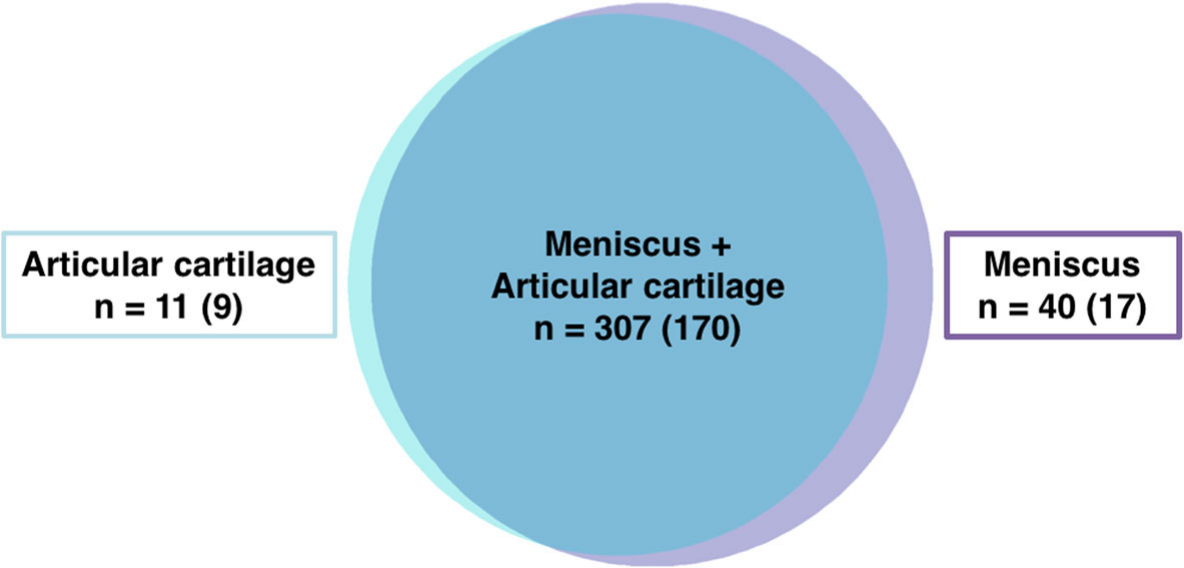
Venn diagram displaying the distribution of proteins in meniscus and articular cartilage. Only proteins that had at least two unique peptides and could be identified in at least three of the samples were included in the figure. The majority of the proteins are common between the two tissues. The numbers for extracellular proteins are shown in brackets

A total of 673 proteins were identified in the DDA analysis, of which 358 proteins had at least two unique peptides and could be identified in at least three of the samples. A full list of these 358 proteins can be found in Additional file 2: Table S1. Out of these, 307 were common between meniscus and articular cartilage, while 40 proteins were unique to the meniscus samples and 11 proteins were specific to articular cartilage (Fig. 1). Out of the 358 proteins, 196 were classified as extracellular matrix (ECM), of which 170 were common between meniscus and articular cartilage. Among the ECM proteins, 17 proteins were found in meniscus only (e.g. collagen IV, both alpha 1 and 2 chains, high mobility group protein B1 and nidogen-2) and 9 proteins in articular cartilage alone (e.g. collagen XI, both alpha 1 and 2 chains, matrilin-3 and serine protease HTRA3) (Fig. 1).

### Protein abundance based on DDA and DIA

**Fig. 2:**
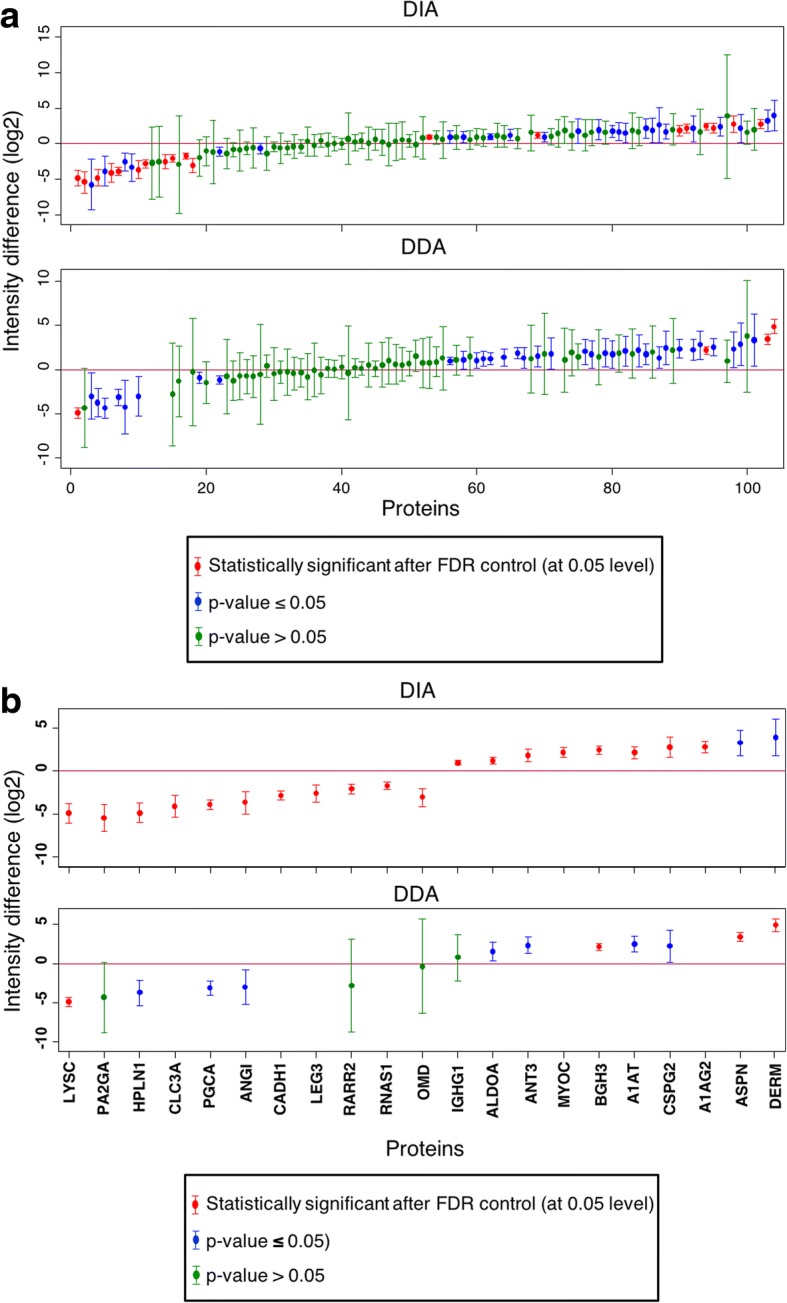
Intensity differences between meniscus and articular cartilage with DIA and DDA. Intensity differences on log2 scale, displayed with 95% confidence intervals, between meniscus and articular cartilage in DDA and DIA, for (**a**) all 90 proteins included in the statistical analysis and (**b**) for the proteins that were statistically significantly different in at least one of the methods

The majority of the included ECM proteins had similar abundance in meniscus and articular cartilage, both in the DDA and DIA analysis (Fig. 2a). However, DIA exhibited more statistically significant proteins and narrower confidence intervals than DDA. Out of 103 proteins 21 differed statistically significantly between the two tissue types in the DIA analysis and 19 remained different after FDR control (Table 1). However, in the DDA analysis, only 11 out of 103 proteins were statistically significantly different and four of these remained after FDR control. Only two proteins, lysozyme C and transforming growth factor-beta-induced protein ig-h3 (BGH3), were statistically significantly different (after FDR control) between the two tissue types in both analysis methods (Fig. 2b). On average the ratios of protein abundance between articular cartilage and meniscus were similar even if numerical differences were found (see Additional file 3*:* Figure S2). For example, among the proteins with higher levels in articular cartilage, phospholipase A membrane-associated and lysozyme C yielded the largest differences in the DIA analysis and DDA analysis, respectively. Among the proteins with higher levels in meniscus, dermatopontin had the largest ratio in both analyses. Still, the DDA approach appeared to yield higher estimated intensity values than DIA as depicted in Additional file 3: Figure S2.

**Table 1:** The statistically significantly different proteins displayed with meniscus (M) vs articular cartilage (C) ratios together with 95% confidence intervals

Protein		DIA		DDA	
Protein name	Entry name	M vs C Ratio	95% CI	M vs C Ratio	95% CI
Phospholipase A2, membrane associated	PA2GA	**0.02**	0.0080, 0.0682	0.05	0.0022, 1.0759
Lysozyme C	LYSC	**0.03**	0.0155, 0.0749	**0.03**	0.0225, 0.0491
Link protein 1	HPLN1	**0.03**	0.0157, 0.0780	**0.07**	0.0244, 0.2262
C-type lectin domain family 3 member A	CLC3A	**0.06**	0.0235, 0.1404	–	–
Aggrecan core protein	PGCA	**0.07**	0.0437, 0.1003	**0.11**	0.0607, 0.2088
Angiogenin	ANGI	**0.08**	0.0322, 0.1914	**0.12**	0.0262, 0.5713
Osteoadherin	OMD	**0.12**	0.0592, 0.2396	0.81	0.0124, 53.1158
Cadherin-1	CADH1	**0.14**	0.0970, 0.1985	–	–
Galectin-3	LEG3	**0.17**	0.0850, 0.3358	–	–
Retinoic acid receptor responder protein 2	RARR2	**0.24**	0.1616, 0.3465	0.14	0.0024, 8.3010
Ribonuclease pancreatic	RNAS1	**0.31**	0.2257, 0.4193	–	–
Immunoglobulin heavy constant gamma 1	IGHG1	**1.90**	1.5899, 2.2697	1.67	0.2201, 12.6297
Fructose-bisphosphate aldolase A	ALDOA	**2.31**	1.7679, 3.0084	**2.87**	1.2702, 6.5020
Antithrombin-III	ANT3	**3.52**	2.0978, 5.9178	**5.07**	2.4498, 10.4777
Alpha-1-antitrypsin	A1AT	**4.37**	2.7565, 6.9337	**5.66**	2.9101, 11.0077
Myocilin	MYOC	**4.40**	2.9193, 6.6454	–	–
Transforming growth factor-beta-induced protein ig-h3	BGH3	**5.32**	3.7506, 7.5369	**4.43**	3.2893, 5.9672
Versican core protein	CSPG2	**6.64**	3.0219, 14.5711	**4.71**	1.1596, 19.1601
Alpha-1-acid glycoprotein 2	A1AG2	**6.83**	4.3899, 10.6362	–	–
Asporin	ASPN	**9.40**	3.3169, 26.6299	**10.79**	7.3853, 15.7771
Dermatopontin	DERM	**14.97**	3.3437, 66.9949	**29.25**	16.3595, 52.2832

Statistically significant ratios are marked in bold

### Top 10 proteins in each tissue type

Furthermore, a descriptive comparison of the 10 proteins with the highest (top 3) peak area intensity representing the most abundant proteins in extracts from articular cartilage and meniscus using DDA was made (Table 2*).* Aggrecan was the protein with the highest intensity in articular cartilage, whereas in meniscus it was serum albumin. Aggrecan was also one of the statistically significantly different proteins displayed in Fig. 1b.

**Table 2:** Top 10 proteins with the highest intensity in the DDA analysis in articular cartilage and meniscus

Rank	Articular cartilage		Meniscus	
	Protein	Mean abundance top 3 peptides	Protein	Mean abundance top 3 peptides
1	Aggrecan core protein	1.47 × 10^10^	Serum albumin	2.17 × 10^10^
2	Cartilage oligomeric matrix protein	1.3 × 10^10^	Decorin	1.37 × 10^10^
3	Decorin	1.27 × 10^10^	Prolargin	1.35 × 10^10^
4	Serum albumin	8.7 × 10^9^	Cartilage oligomeric matrix protein	1.12 × 10^10^
5	Prolargin	7.83 × 10^9^	Mimecan	8.27 × 10^9^
6	Fibronectin	7.33 × 10^9^	Biglycan	5.63 × 10^9^
7	Fibromodulin	6.53 × 10^9^	Lumican	5.3 × 10^9^
8	Biglycan	5.4 × 10^9^	Fibromodulin	5.1 × 10^9^
9	Cartilage intermediate layer protein 1	4.47 × 10^9^	Hemoglobin subunit beta	3.43 × 10^9^
10	Mimecan	3.6 × 10^9^	Clusterin	3.27 × 10^9^

## Discussion

Articular cartilage remains the most studied tissue in OA, but the knowledge about the meniscus is still limited. In this study, a comparison of knee articular cartilage and meniscus was made in order to gain new knowledge of its composition and also to evaluate two analytical methods for MS: DDA and DIA. We identified several differences between the articular cartilage and meniscus proteome.

Approximately 14% of the proteins were identified in either meniscus or articular cartilage (Fig. 1) alone, which could be due to biological differences and sample complexity e.g. the vascularization of the meniscus is likely to impact the results allowing more plasma proteins to be present and thereby detected. Many of the identified proteins could be highly relevant for the tissue characterisation in OA, but since one aim of the study was to compare the two MS methods by statistical means we only included proteins that were identified in both tissue types.

### Single protein comparisons

Even though the majority of the selected proteins did not substantially differ in abundance between meniscus and articular cartilage, we detected distinct differences for certain specific proteins. However, lysozyme C was one the proteins that were significantly different between the tissue types, after FDR control, in both the DDA and DIA analysis (Fig. 2b). Similarly, lysozyme C was previously reported to be more abundant in articular cartilage than meniscus [8, 17]. Already discovered by Flemming in the 1920s, lysozymes are characterized as cationic proteins that primarily has a bacteriolytic function, and their presence in articular cartilage has been suggested to be due to ionic interactions with high levels of anionic aggrecan that would enable protein enrichment [18–20]. Indeed, aggrecan was found to have a lower abundance in meniscus, which could explain the lower abundance of lysozyme C. The exact function of lysozyme C in articular cartilage remains unknown, however it is possible that lysozyme C is part of the intra-articular defence against bacteria [21].

The other protein that was statistically significantly different in abundance between meniscus and articular cartilage, after FDR control, was BGH3 (Fig. 2b). We found that BGH3 had a 5-fold higher expression in meniscus than articular cartilage. BGH3 is known to be expressed in several tissues and has been suggested to have a negative effect on chondrogenesis [22]. Although it is not known exactly how BGH3 is incorporated into the ECM, it has been observed to bind to several collagens as well as fibronectin, decorin and biglycan [22, 23].

Small leucine-rich proteoglycans (SLRPs) is a group of highly abundant ECM proteins in cartilage. They bind to several collagens and have been reported to have effect on various cellular functions, e.g. due to their ability to bind numerous cell surface receptors and growth factors, and in later years they have also been associated with OA pathogenesis [24]. The group of SLRPs includes 18 members [25], of which 8 were included in this analysis. Asporin, at the protein level, was first described by Lorenzo et al. in 2001 [26], and has been reported to be able to inhibit TGFß-mediated chondrogenesis [27, 28]. In this study, asporin was found to be more abundant in meniscus than in articular cartilage, both in the DDA and DIA analysis with meniscus to articular cartilage ratios of 10.79 and 9.40 respectively (Table 1). A previous study also reported an enrichment of asporin in the meniscus compared to articular cartilage [8]. Two other members of the SLRP family that exhibited similar trends as asporin in this study was decorin and biglycan, of which both were among the top 10 proteins in both articular cartilage and meniscus (Table 2). Both proteins had a higher intensity in meniscus, however this was not statistically significant.

Aggrecan is the most abundant proteoglycan in articular cartilage and plays a very important role to trap water and make the articular cartilage able to withstand compressive load. In this analysis, the aggrecan level was approximately 10 times higher in articular cartilage than in meniscus (Table 1). This is consistent with previous studies, which have reported that aggrecan mRNA levels are lower in human meniscus than articular cartilage and that total proteoglycan synthesis is lower in bovine fibrochondrocytes of the meniscus than articular chondrocyte-populated constructs [29, 30]. Furthermore, most of the aggrecan is bound to hyaluronic acid and link proteins [31], which is probably why the proteoglycan link protein 1 (HPLN1) correlate with aggrecan (Fig. 2b). In contrast, the proteoglycan versican was more abundant in meniscus, both in DDA and DIA (Table 1). Versican was first described as a fibroblast proteoglycan [32], and in one study comparing aggrecan and versican mRNA levels in chondrocytes and fibroblasts, it was observed that versican mRNA levels were higher in fibroblasts than chondrocytes, while aggrecan could only be detected in chondrocytes [33]. This could explain the higher abundance of versican in meniscus since it contains both chondrocyte-like cells and fibroblast-like cells [6]. Aggrecan and versican belong to the same family of hyaluronan-binding proteoglycans [34], and while the function of aggrecan in articular cartilage is well-known, more research is needed to fully elucidate the role versican plays in the meniscus.

The protein with the highest significant intensity difference in meniscus compared to articular cartilage in this study was dermatopontin, with a meniscus to articular cartilage ratio of approximately 15 and 29 in the DIA and DDA analysis respectively (Table 1). Dermatopontin is an ECM protein that has been associated with promotion of cell attachment and spreading of dermal fibroblasts [35] and has been reported to have a higher expression in healthy menisci than OA menisci using proteomics [36]. The presence of fibroblast-like cells in the outer regions of menisci could explain the increased intensity of dermatopontin in our meniscus samples [37].

### Vascularisation

As previously described, the meniscus is partly vascularized, while articular cartilage is avascular [38, 39]. In this study, we can report that among the proteins with at least two unique peptides, 25 can be classified as blood circulation proteins using the GO accession GO:000815. Of these, 8 were found to be meniscus-specific and none were unique to articular cartilage. Furthermore, serum albumin is the protein with the highest intensity in meniscus in the DDA analysis (Table 2), which further supports the previous reports of a vascularized meniscus. The list of the ten most abundant proteins in meniscus also contains two additional well-known plasma proteins; haemoglobin subunit beta and serotransferrin (Table 2). One protein that significantly differed between meniscus and articular cartilage was alpha-1-antitrypsin (A1AT). This protein is one of the top 20 most abundant proteins in plasma, and it is therefore not surprising that A1AT is on average approximately five times more abundant in meniscus than articular cartilage (Table 1). However, several plasma proteins were identified also in articular cartilage in this study. Since articular cartilage is in contact with synovial fluid in the joint, and synovial fluid is an ultra-filtrate of plasma and therefore contains plasma proteins, the presence of plasma proteins in articular cartilage might be explained by the ability of the cartilage to absorb molecules, in this case plasma proteins, from its surroundings. This could for example be the case for angiogenin, a protein found in the circulation and reported to be angiogenic [40]. It was significantly more abundant in articular cartilage than meniscus in this study. Angiogenin is a 14 kDa and 123 amino acids long protein that is highly cationic [41, 42], making it similar to lyzosyme C in both size and charge [17]. These common traits with lysozyme C, which is enriched in articular cartilage, could explain the presence of angiogenin in articular cartilage despite its avascular phenotype. Similarly, in one study, the authors reported that angiogenesis might be involved in OA, and hypothesised that articular cartilage might be able to take up circulating molecules, e.g. from the vascularised synovium and that there was an invasion of synovium into the articular cartilage [43]. Furthermore, in a study investigating the role of angiogenesis in hip OA, it was noted that the grade of angiogenesis was related to the cartilage degeneration, hence it could be involved in the degenerative process [44].

### Data-dependent acquisition vs data-independent acquisition

The second aim of this study was to compare DDA and DIA in order to decide which method to use in future studies. First of all, DIA yielded more precise estimates than DDA. As a consequence, many proteins in the DIA analysis were also statistically significant after FDR control, while only a few in the DDA analysis. In addition to this, DIA had no missing values. Out of the 103 proteins that remained after peak selection in Skyline and selected for statistical analysis, we removed 13 from the analysis due to missing values in the DDA analysis. This is due to the fact that DDA randomly measures only the most abundant peptides if too many peptides elute at the same time in one MS1 scan, then the low abundant peptides are therefore missed [45]. This makes DDA data less reproducible than DIA data, and there is also a risk that the low-abundant proteins are excluded [45]. This problem is circumvented in DIA, since all precursor ions within a certain m/z range are measured [9, 13]. Another advantage with DIA is that it is possible to base the quantitation on MS2, which is not possible with DDA. MS2 is also often used for identification and validation with DIA, which is only possible with DDA if MS2 is available. One factor that might be regarded as a disadvantage with DIA is that it is more time-consuming than DDA, as the manual peak selection that needs to be performed before the data can be analysed is tedious [45]. Another feature is that the DIA data contains more complex MS2 spectra that require a spectral library for extracting the data. Improved software solutions could have a great impact on these drawbacks. The high complexity of the data also results in the need of larger data storage space, which might be a challenge. Even though DIA might be superior to DDA in several ways, on average, both methods, as expected, give similar point estimates of the differences between the two analysed tissues. The higher precision of DIA can probably be explained by the fact that the peptides are manually selected in DIA, resulting in the removal of peptides with worse chromatographic performance. Taken together, the advantages of DIA make it the preferred method of choice.

### Limitations

Since this was a pilot analysis, the sample size was limited; hence resulting in a lower power and a higher risk of type 2 errors, i.e. the comparison of single proteins between the tissues should be interpreted with caution. Furthermore, we chose to narrow down the number of included proteins in order to make the analyses less complex e.g. as OA is a disease that markedly affects the ECM, therefore only ECM proteins were included in the analyses. The ECM proteins were filtered by the GO accession term GO:0031012. There are some limitations connected with the usage of GO accession terms e.g. the fact that there are several terms that refer to ECM proteins and that there is a possibility that some ECM proteins might have been lost in the filtration. However, we chose the one we thought would be most suitable for this study and the most common ECM proteins have been included in the analysis, which is sufficient since the main aim of the filtration was to select a number of proteins to include in our method comparison.

## Conclusions

Despite similarities in protein expression between articular cartilage and meniscus, 21 proteins differed between the two tissues in the DIA analysis. Eleven out of these also differed in the DDA analysis. In articular cartilage, aggrecan core protein was the most abundant protein and phospholipase A membrane-associated and lysozyme C had the largest articular cartilage to meniscus ratio in the DIA and DDA analysis respectively. In meniscus, serum albumin was the most abundant protein and dermatopontin had the largest meniscus to articular cartilage ratio. More research is needed to fully elucidate the molecular mechanisms behind these differences in protein expression. Comparing the two methods DIA has several clear advantages over DDA, e.g. no missing values and lower variance, therefore DIA will be our method of choice in future studies in OA research.

## Additional files

Additional file 1: Sample preparation workflow. Schematic representation of the sample preparation steps from tissue collection to (LC)-MS/MS analysis. *GdnHCl* = Guanidine hydrochloride, *AMBIC* = ammonium bicarbonate, *GAG* = glycosaminoglycan, *ACN* = acetonitrile, *LC-MS/MS* = liquid chromatography coupled with tandem mass spectrometry, *DDA* = data-dependent acquisition, *DIA* = data-independent acquisition (TIF 24992 kb)

Additional file 2: Proteins identified with DDA. Table containing all proteins with at least two unique peptides per protein and present in at least three of the samples that were identified in the DDA analysis. The table further indicates if the protein was found exclusively in articular cartilage, meniscus or in both tissues as well as which proteins that were selected for statistical analysis. (XLSX 42 kb)

Additional file 3: Agreement analysis of DDA and DIA results. The estimates are ratios (DIA vs DDA) of intensity ratios between meniscus and cartilage, with 95% confidence intervals. A ratio of 1 indicates that the protein intensity ratios from DIA and DDA methods were equal, and is marked with a red line. (DOCX 226 kb)

## Abbreviations

DDA: Data-dependent acquisition
DIA: Data-independent acquisition
ECM: Extracellular matrix
FDR: False discovery rate
LC: Liquid chromatography
MS: Mass spectrometry
OA: Osteoarthritis
