# Supplementary figures and images for: Differential protein expression in human knee articular cartilage and medial meniscus using two different proteomic methods: a pilot analysis
Source: BMC Musculoskelet Disord. 2018 Nov 29;19:416. doi: 10.1186/s12891-018-2346-6 (PMC6267052; doi:10.1186/s12891-018-2346-6)

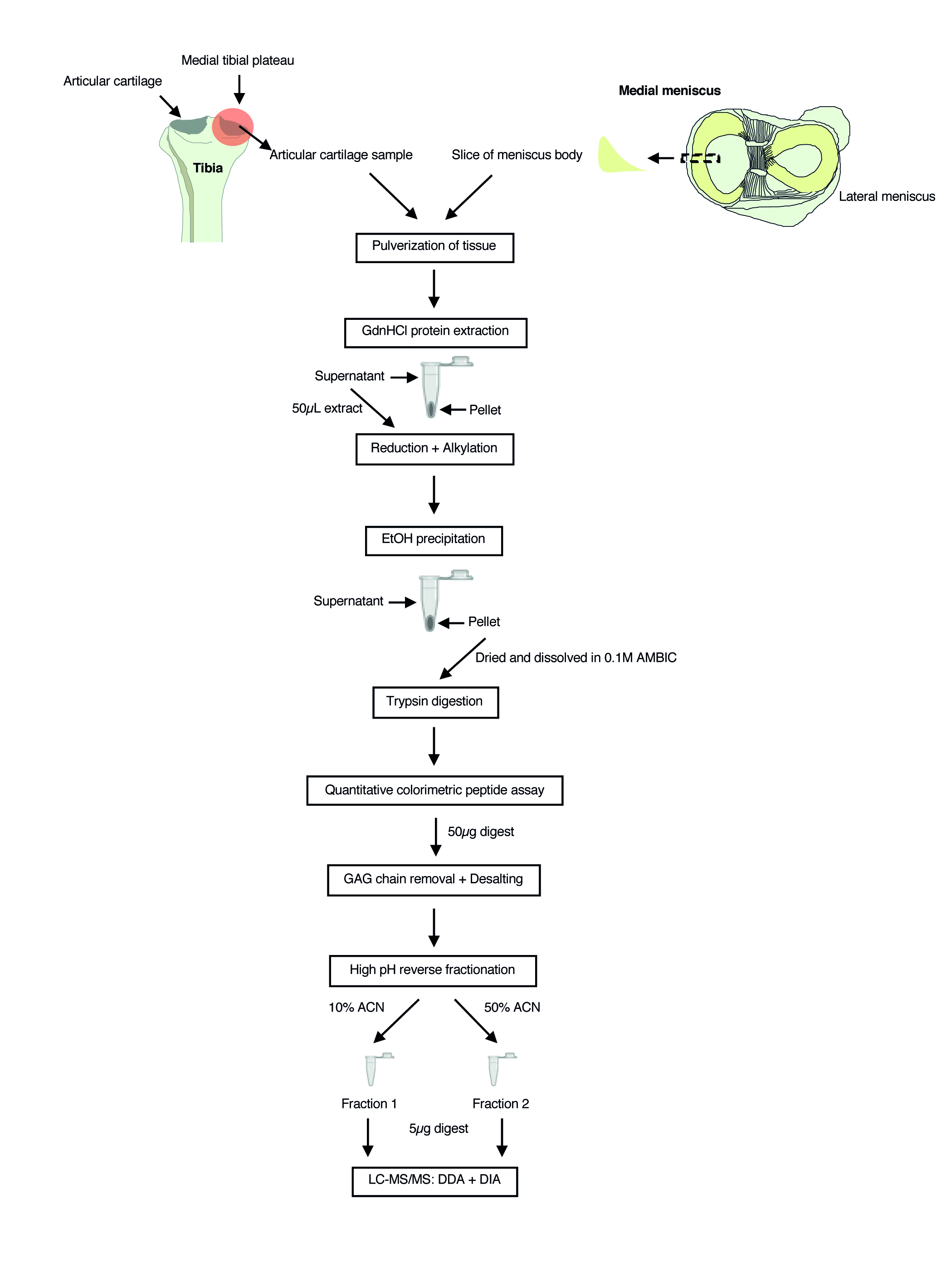

Supplement: Supplementary file 1 — Sample preparation workflow. Schematic representation of the sample preparation steps from tissue collection to (LC)-MS/MS analysis. GdnHCl = Guanidine hydrochloride, AMBIC = ammonium bicarbonate, GAG = glycosaminoglycan, ACN = acetonitrile, LC-MS/MS = liquid chromatography coupled with tandem mass spectrometry, DDA = data-dependent acquisition, DIA = data-independent acquisition (TIF 24992 kb) [file 12891_2018_2346_MOESM1_ESM.tif]

**Supplementary fig. 2**

**
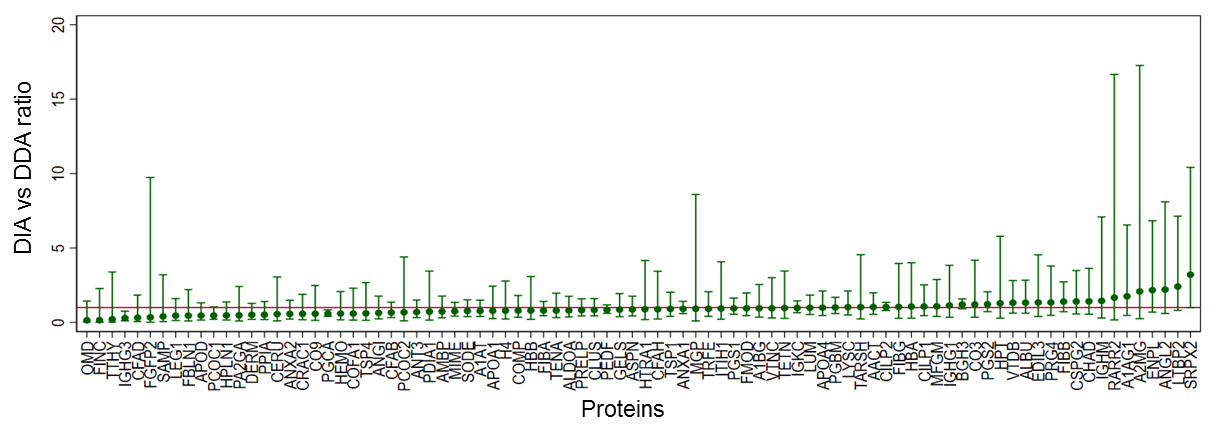
**

Supplement: Supplementary file 3 — Agreement analysis of DDA and DIA results. The estimates are ratios (DIA vs DDA) of intensity ratios between meniscus and cartilage, with 95% confidence intervals. A ratio of 1 indicates that the protein intensity ratios from DIA and DDA methods were equal, and is marked with a red line. (DOCX 226 kb) [file 12891_2018_2346_MOESM3_ESM.docx]
